# What Do Transgender Patients Teach Us About Idiopathic Intracranial Hypertension?

**DOI:** 10.1080/01658107.2017.1316744

**Published:** 2017-05-10

**Authors:** Catherine Hornby, Susan P. Mollan, James Mitchell, Keira Annie Markey, Andreas Yangou, Ben L. C. Wright, Michael W. O’Reilly, Alexandra J. Sinclair

**Affiliations:** a Institute of Metabolism and Systems Research, University of Birmingham, Edgbaston, United Kingdom; b Centre for Endocrinology, Diabetes and Metabolism, Birmingham Health Partners, Birmingham, United Kingdom; c Department of Neurology, University Hospitals Birmingham NHS Foundation Trust, Birmingham, United Kingdom; d Birmingham Neuro-Ophthalmology Unit, Department of Ophthalmology, University Hospitals Birmingham NHS Trust, Queen Elizabeth Hospital Birmingham, Birmingham, United Kingdom

**Keywords:** Androgens, gender reassignment, idiopathic intracranial hypertension, papilloedema testosterone

## Abstract

Idiopathic intracranial hypertension (IIH), a condition of raised intracranial pressure, is characterised by headaches and visual disturbances. Its pathogenesis is currently unknown; however, dysregulation of androgens may be implicated. Here, the authors present a case of a 22-year-old patient undergoing female-to-male (FTM) gender reassignment who developed IIH shortly after commencing testosterone therapy. This interesting case presents the possibility of androgens having a pathogenic role in IIH.

Idiopathic intracranial hypertension (IIH) is a disorder of raised intracranial pressure (ICP), typically presenting with headaches, papilloedema, and visual loss. Headaches can be severe, causing significant morbidity, although there is increasing recognition of the variability in their presentation.^1^ There is also a recognised subset of patients where papilloedema is not present, IIH without papilloedema (IIHWOP).^2^ The condition has a predilection for obese women of childbearing age,^3^ yet the reasons for this are not clear.^4^

A condition with similar phenotypic characteristics of obese, young women is polycystic ovary syndrome (PCOS). Androgen excess is a defining feature of PCOS.^5^ There is an increased prevalence of PCOS in the IIH population, with reports of up to 57% of IIH patients also having PCOS,^6^ compared with a prevalence of 5–10% in the general population.^7^ It is interesting to speculate that IIH, akin to PCOS, could be driven by androgen excess. In support of this, a study has found that hyperandrogenism is linked with younger age of onset of IIH but is not associated with body mass index (BMI), waist-to-hip ratios, or duration of IIH.^8^

Karyotypically female patients undergoing female-to-male (FTM) gender reassignment with testosterone therapy offer an important opportunity to increase our understanding of the role of androgens in IIH. Here, we present the case of a patient undergoing gender reassignment who developed IIH on commencing testosterone therapy.

## Case report

**Figure 1.**
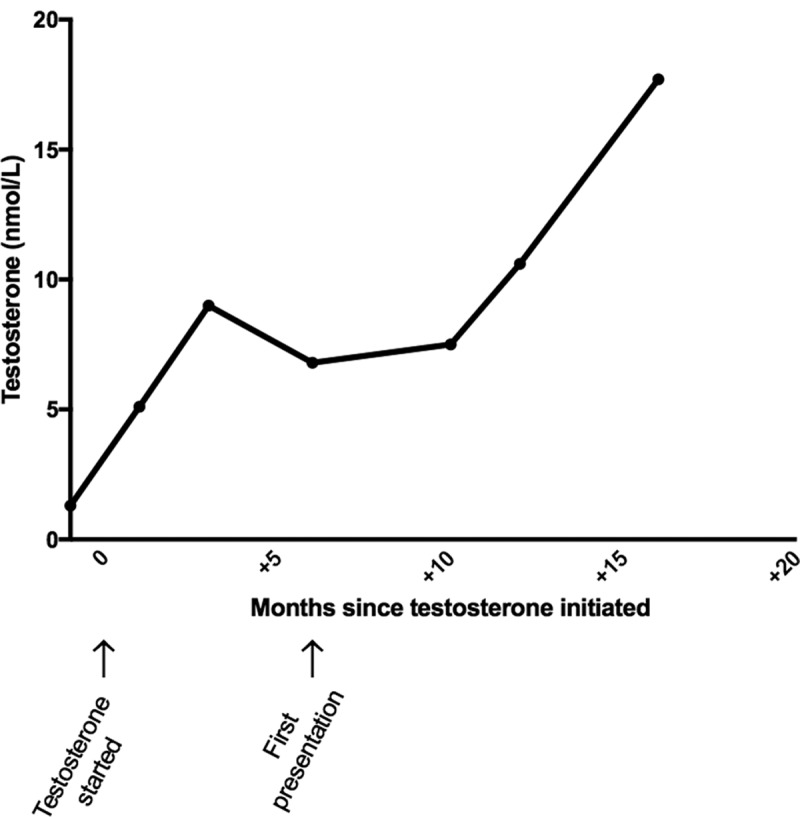
Profile of serum testosterone over time since initiation of therapy, with first presentation at 6 months.

**Figure 2.**
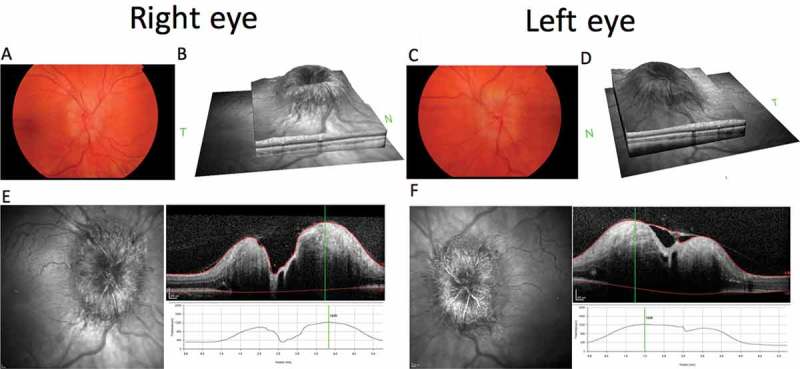
Composite figure showing colour fundus photographs of the papilloedema affecting the optic nerve head (OHN) of the right (A) and left (C) eyes. Optical coherence tomography (OCT) SPECTRALIS HRA+OCT (Heidelberg Engineering, Heidelberg, Germany), infrared (IR) images of the ONH, and volume cross-sectional images and the elevated height through the centre of the ONH of the right (E) and left (F) eyes. OCT IR disc volume reconstructions for right (B) and left (D) eyes.

A 22-year-old karyotypically 46XX patient undergoing FTM gender reassignment presented with a 6-month history of new daily headaches and 5 months of intermittent visual blurring, pulsatile tinnitus, and nausea. He had started testosterone injections (250 mg 3-monthly) just prior to symptom onset (Figure 1). His visual symptoms progressed, and he began to develop transient visual obscurations up to 50 times per day. He presented to his opticians, who noted bilateral papilloedema and referred him to neuro-ophthalmology. Best-corrected visual acuity was 6/9 bilaterally, Ishihara colour vision was normal, and there was no relative afferent pupillary defect (RAPD). He was confirmed to have severe bilateral papilloedema, modified Frisén grade 3 in the right eye and grade 4 in the left eye,^9^ with no sixth nerve palsy. Spectral-domain optical coherence tomography was performed (Figure 2), and the overall maximum retinal nerve fibre layer (RNFL) height was 1226 μm for the left eye and 1218 μm for the right eye. Goldmann visual fields showed bilateral increase in the blind spot, loss of some static central points in the right eye, with the left eye showing an increased nasal step. Magnetic resonance (MR) imaging of the brain (Siemens 3.0 Tesla) showed a partial empty sella on T2-weighted imaging, and MR venography demonstrated no cerebral sinus thrombosis. Two lumbar punctures a few days apart recorded opening pressures of 40 and 39 cm cerebrospinal fluid (CSF). Although he was classed as overweight on starting testosterone injections (BMI 27.9 kg/m^2^), his weight had only increased over the period of the testosterone therapy to a very small degree (+3.3 kg in 6 months). His full blood count, renal and liver functions, and inflammatory markers were normal. His regular medications consisted of regular pain relief with codeine and naproxen, zopiclone 7.5 mg, sertraline 50 mg OD, desogestrel 75 μg (for suppression of menses and endometrial protection), and 3-monthly testosterone injections (250 mg). There was no evidence of other secondary cause of raised ICP. Due to emergent deterioration in visual function, he was referred for CSF diversion with a lumbo-peritoneal shunt.

Following lumbo-peritoneal shunt insertion, papilloedema settled. The patient decided to continue testosterone injections; the testosterone levels remained elevated (Figure 1) and his weight remains stable. At 18 months post shunt insertion, the IIH remained in remission and he went on to have a bilateral mastectomy.

## Discussion

This case is unusual, as it reports the development of IIH in a transgender patient on testosterone therapy. The temporal relationship of initiation of testosterone therapy and the onset of symptoms in this case may suggest a link between raised intracranial pressure and testosterone. However, this occurrence is not in isolation, as four other reports in the literature document similar cases of IIH arising in patients undergoing gender reassignment with testosterone therapy, with three having a temporal relationship with commencing testosterone therapy.^10,11,13^ All cases reported were reassigning their gender from female to male (Table 1).

**Table 1.** Summary of cases IIH in gender reassignment in the literature to date.

Author	Year	Gender reassignment	Symptom occurrence	Testosterone temporally causative	Body mass index (kg/m^2^)	Treatment	Outcome
Case presented	2017	FTM	Symptoms correlate with testosterone commencing	Yes	27.9	LP shunt	Remission of raised intracranial pressure
Buchanan et al.^11^	2017	FTM	Increasing dose of testosterone, and increased weight	Yes	Not known	Treatment was initially reducing testosterone hormone therapy by 50% and acetazolamide. Then subsequent weight loss	Remission
Park et al.^13^	2014	FTM	Patient was on testosterone	Yes	Not known	Swapped to longer-acting testosterone therapy and started acetazolamide	Remission
Mowl et al.^10^	2009	FTM	Patient was on testosterone	Yes	27	Reducing testosterone therapy and Diamox	Remission
Sheets et al.^12^	2007	FTM	Symptoms started 10 months *after* discontinuing testosterone	No	29.8	Initially acetazolamide, due to side effects switched to frusemide and topiramate. Subsequent unilateral optic nerve sheath fenestration	Remission

Transgender patients who develop IIH provide an insight into the potentially pathogenic role of testosterone in IIH. The predilection of the disease for a particular gender would suggest that hormones may play a role in pathogenesis; however, previous studies into the role of oestrogens were inconclusive and have not been replicated.^14,15^ It may be that the class of hormones that need further investigation are the androgens, as highlighted by this case report and the previous cases (Table 1), coupled with the observation that another androgen dysregulated condition, PCOS, shares a strikingly similar clinical phenotype. Furthermore, one study reports an increased incidence (58%) of PCOS in FTM patients prior to hormonal therapy.^16^ It is notable that IIH has also been reported in men with hypogonadism, with cases described in men after androgen deprivation therapy for prostate cancer.^17^ Additionally, men with IIH are more likely to have symptoms associated with testosterone deficiency.^18^ This raises the prospect of a “pathophysiological window” of circulating testosterone levels in humans, with levels above the normal reference range for females, but below the normal reference range for men, associated with metabolic perturbations, including increased visceral fat deposition, insulin resistance, and fatty liver disease.^19^ Serum testosterone levels in the cases described above were within this window when first presentation occurred. It is therefore feasible that IIH is a distinct neuro-metabolic complication of circulating testosterone levels within this range. These observations present an interesting avenue for new research in IIH and warrant the need for further detailed characterisation of the androgen metabolic phenotype in this condition.
